# Functional and Pathological Roles of AHCY

**DOI:** 10.3389/fcell.2021.654344

**Published:** 2021-03-31

**Authors:** Pedro Vizán, Luciano Di Croce, Sergi Aranda

**Affiliations:** ^1^Centre for Genomic Regulation (CRG), Barcelona Institute of Science and Technology, Barcelona, Spain; ^2^Universitat Pompeu Fabra (UPF), Barcelona, Spain; ^3^Catalan Institution for Research and Advanced Studies (ICREA), Barcelona, Spain

**Keywords:** adenosylhomocysteinase, *S*-adenosylhomocysteine hydrolase, *S*-adenosylmethionine, gene regulation, epigenetics, chromatin, embryo development

## Abstract

Adenosylhomocysteinase (AHCY) is a unique enzyme and one of the most conserved proteins in living organisms. AHCY catalyzes the reversible break of *S*-adenosylhomocysteine (SAH), the by-product and a potent inhibitor of methyltransferases activity. In mammals, AHCY is the only enzyme capable of performing this reaction. Controlled subcellular localization of AHCY is believed to facilitate local transmethylation reactions, by removing excess of SAH. Accordingly, AHCY is recruited to chromatin during replication and active transcription, correlating with increasing demands for DNA, RNA, and histone methylation. AHCY deletion is embryonic lethal in many organisms (from plants to mammals). In humans, AHCY deficiency is associated with an incurable rare recessive disorder in methionine metabolism. In this review, we focus on the AHCY protein from an evolutionary, biochemical, and functional point of view, and we discuss the most recent, relevant, and controversial contributions to the study of this enzyme.

## Introduction

**FIGURE 1:**
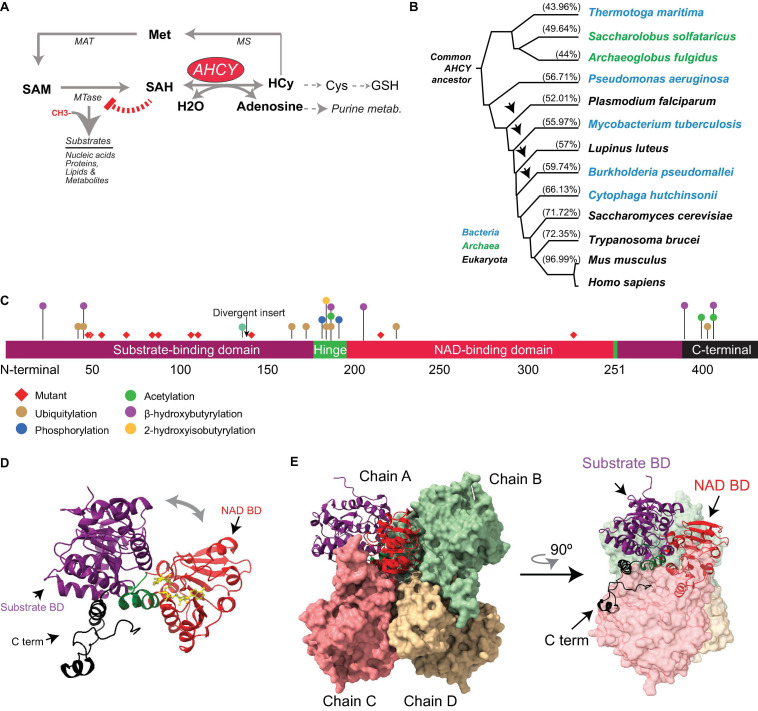
Evolution and structure of adenosylhomocysteinase (AHCY). **(A)** Scheme of the methionine (Met) metabolic pathway. Enzymes of the pathway are indicated AHCY, methionine adenosyltransferase (MAT), methionine synthase (MS), and SAM-dependent methyltransferase (MTase). MTase transfer a methyl group from SAM to substrates, thereby generating SAH. AHCY breaks SAH into adenosine and homocysteine (Hcy). Hcy can be recycled to methionine (Met) coupled to folate metabolism or to produce glutathione. **(B)** Selected AHCY amino acid sequences were obtained from Uniprot (https://www.uniprot.org/) using the following accession numbers *Thermotoga maritima* (O51933), *Saccharolobus solfataricus* (P50252), *Archaeoglobus fulgidus* (O28279), *Pseudomonas aeruginosa* (Q9I685), *Plasmodium falciparum* (P50250), *Mycobacterium tuberculosis* (P9WGV3), *Lupinus luteus* (Q9SP37), *Burkholderia pseudomallei* (Q3JY79), *Cytophaga hutchinsonii* (A0A6N4SNR7), *Saccharomyces cerevisiae* (A0A6A5PY71) *Trypanosoma brucei* (Q383X0), *Mus musculus* (P50247), and *Homo sapiens* (P23526). The tree was generated by Clustal Omega (https://www.ebi.ac.uk/Tools/msa/clustalo/) and Phylodendron (http://iubioarchive.bio.net/treeapp/treeprint-form.html). The % indicates the similarity with human protein (P23526) calculated using BLAST (https://blast.ncbi.nlm.nih.gov/Blast.cgi). The arrow indicates the species where the ∼40-amino acid insert is present. **(C)** The different functional modules of human AHCY are represented. The numbers indicate the position of amino acids. The arrow indicates the position of the divergent insert found in some bacteria and eukaryotes, including plants. Red diamonds indicate the position of the twelve mutations founds in patients with AHCY deficiency (R49C, R49H, A50T, T57I, G71S, D86G, A89V, E108K, T112stop, Y143C, V217M, and Y328D). The colored dots indicate the position of posttranslational modifications found in PhosphoSitePlus, with at least five references, and in the literature. **(D)** Structural model for the human AHCY monomer in the open conformation, the co-factor is depicted in yellow in association with the NAD-binding domain (Protein Data Bank-PDB: 4yvf). The different modules are colored as in **(C)**. **(E)** Structural model of human AHCY tetrameric enzyme in close conformation (protein data bank-PDB: 3nj4). The different modules are colored as in **(C)**.

The metabolic enzyme adenosylhomocysteinase (AHCY; also alternatively called SAHH for *S*-adenosyl-L-homocysteine hydrolase, according to HUGO Gene Nomenclature Committee) is one of the most conserved enzymes in living organisms, including bacteria, nematodes, yeast, plants, insects, and vertebrates (Kusakabe et al., 2015). In mammals, AHCY is the only enzyme that mediates the reversible catalysis of *S*-adenosylhomocysteine (SAH) to adenosine and L-homocysteine (Turner et al., 1998) (Figure 1). AHCY acts within the one-carbon metabolic cycle, a universal metabolic process that enables the transfer of one-carbon units for biosynthetic processes (purines and thymidine), amino acid homeostasis (cysteine, serine, and methionine), redox cellular control, and epigenetic regulation (Ducker and Rabinowitz, 2017). SAH is the product of *S*-adenosyl-L-methionine (SAM)-dependent methyltransferases (MTases), which transfers the methyl group from SAM to a variety of cellular substrates, including nucleic acids and proteins.

*S*-adenosyl-L-methionine is the major methyl donor in the cell and the second most widely used cofactor after ATP (Clarke, 2001; Ye and Tu, 2018). SAH is structurally similar to SAM and binds with a similar range of affinity to the SAM-binding pocket of MTases, thereby inhibiting their activity in a negative feedback loop (Clarke, 2001; Richon et al., 2011; Zhang and Zheng, 2016) (Figure 1). As SAM and SAH compete for the SAM-binding pocket of MTases, the SAM:SAH ratio is considered an indicator of the methylation capacity of cells, and its decrease can correlate with a reduction of the methylation potential (Clarke, 2001; Petrossian and Clarke, 2011).

Unlike acetylation, which only occurs on proteins, methylation can be found at DNA, RNA, and proteins (Clarke, 2001). Chromatin is a macromolecular complex formed by DNA, together with physically associated proteins and non-coding RNAs (Espejo et al., 2020). Nucleosomes are the functional unit of the chromatin and comprise two of each of the core histones H2A, H2B, H3, and H4, which wrap up ∼147 base pairs of DNA. Chemical modifications on DNA and histones modulate gene expression and, in some instances, are responsible for conveying the epigenetic information to control cell-type-specific gene expression programs across cell division (Bonasio et al., 2010; Aranda et al., 2015). Indeed, the potential impact of AHCY in controlling methylation of DNA, RNA, and histones has been studied in many model organisms and cell types.

Here, we review the evolutionary conservation of AHCY across living organisms, its enzymatic structure, and its catalysis. We also detail the functional and molecular roles of AHCY in cellular homeostasis in different model organisms. Finally, we discuss the pathogenic influence of AHCY in human disease.

## Evolution, Structure, and Regulation of Enzymatic Activity of AHCY

### Evolution

Adenosylhomocysteinase is a highly conserved enzyme present in all three major domains: Archaea, Bacteria, and Eukarya (Tehlivets et al., 2013; Kusakabe et al., 2015) (Figure 1). Comprehensive phylogenetic analyses suggest that AHCY evolved from a common ancestor in Archaea and Eukarya, and that orthologs transferred horizontally from these clades to bacteria lineage multiple times during evolution (Bujnicki et al., 2003; Stepkowski et al., 2005). In the absence of AHCY, many bacterial species (e.g., *Escherichia coli*) rely on the 5′-methylthioadenosine/*S*-adenosylhomocysteine (MTA/SAH) nucleosidase (which is lacking in mammals) for SAH withdrawal (Stepkowski et al., 2005).

The evolutionary reconstruction of the AHCY protein highlights two major divergences in its sequence. First, an insertion of about 40 amino acids is present within the catalytic domain in many bacteria and some eukaryotes, including plants and some pathogens (such as *Plasmodium falciparum*). In contrast, this insertion is absent Archaea and several eukaryotes, including model organisms such as *Dictyostelium discoideum*, *Saccharomyces cerevisiae*, *Caenorhabditis elegans*, and *Drosophila melanogaster*, as well as vertebrates (Bujnicki et al., 2003; Stepkowski et al., 2005). The insertion does not interfere with the folding of the catalytic domain (Tanaka et al., 2004; Reddy et al., 2008), and rodent insert-less AHCY can functionally restore loss-of-function mutants of *Ahcy* (with insertion) in the purple bacteria *Rhodobacter capsulatus* (Aksamit et al., 1995). Of note, in *Arabidopsis thaliana*, specific deletion of this insertion reveals its role in mediating interactions with proteins, such as adenosine kinase and the mRNA cap MTase, as well as the nuclear localization of AHCY, suggesting a regulatory role for this specific insert in plants (Lee et al., 2012).

The second divergent region in AHCY during evolution is a stretch of about eight amino acids in the *C*-terminal domain present in bacteria and eukaryotes (but not in Archaea), which stabilizes the interaction of AHCY with the NAD^+^ cofactor (Hu et al., 1999; Komoto et al., 2000; Kusakabe et al., 2015). In Archaea, the observed strong affinity for the NAD^+^ cofactor suggests that the lack of a *C*-terminal tail in AHCY must be replaced by other specific residues (Porcelli et al., 1993).

In eukaryotes, AHCY is among the top 100 most-conserved proteins between yeast and mammals, with about 70% of identity (Mushegian et al., 1998). From zebrafish to humans, *AHCY* encompasses 10 exons with nearly identical size, indicating that the high conservation in vertebrates is also maintained at a structural genomic level (Zhao et al., 2009). The human genome harbors one *AHCY* gene in chromosome 20, and 7 pseudogenes in different chromosomes. *AHCY* is ubiquitously expressed in adult tissues (Chen et al., 2010) and encodes three annotated splicing isoforms (AHCY1 (NP_000678), −2 (NP_001155238), and −3 (NP_001309015), with 432, 404, and 434 amino acids, respectively) that differ only in their first 30 amino acids. These isoforms have not been formally compared side-by-side, although ectopically expressed hAHCY1 mutant lacking the first 14 amino acids accumulates in the nucleus and form catalytically dead tetramers (Grbesa et al., 2017). In-depth molecular and functional analyses are now required to verify and then elucidate a functional regulatory role of this *N*-terminal region.

### Structure

Several structures of AHCY have been resolved at an atomic level from bacteria (i.e., *Mycobacterium tuberculosis* and *Pseudomonas aeruginosa*), pathogenic eukaryotes (i.e., *Trypanosoma brucei* and *Plasmodium falciparum*), plants, rodents, and human, all of which share a high structure conservation (Brzezinski, 2020). While some evidence indicates that AHCY can exist in monomeric, homodimeric, and homotetrameric forms within cells (Seo and Ettinger, 1993a,b), all AHCY structures (except that of the plant enzyme *Lupinus luteus*) have been resolved as active homotetramers, with one NAD^+^ cofactor bound in each subunit (Turner et al., 1998; Hu et al., 1999; Brzezinski et al., 2008; Kusakabe et al., 2015). Each monomer comprises three conserved domains (Figure 1): (*i*) a substrate-binding/catalytic domain (SBD; amino acids [aa] 1–181 and 355–385 in human AHCY); (*ii*) a NAD cofactor-binding domain (CBD; aa 197–351); and (*iii*) a *C*-terminal tail (aa 386–432). Both the SBD and the CBD are connected by two hinge regions (aa 182–196 *N*-terminal hinge, and 352–354 *C*-terminal hinge) (Turner et al., 1998; Kusakabe et al., 2015). The central core of the homotetramer is occupied by the cofactor-binding domains from different subunits, and the substrate-binding domains reside at the surface of the tetramer. Notably, the cofactor-binding domain is spatially separated from the substrate-binding domain by a deep cleft in each monomer. The homotetramer is a dimer of dimers, as the *C*-tail domains from two pairs extend and contact, reciprocally, the NAD-binding domain of the other pair (Turner et al., 1998; Yang et al., 2003; Kusakabe et al., 2015). The tetrameric structure is sustained by intersubunit interactions between two alpha helices (aa 184–187 and aa 190–207) from the adjacent subunits (Turner et al., 1998; Yang et al., 2003).

Structural analyses and computational modeling using different resolved structures indicate that each monomer can exist in an “open” (bound to NAD^+^) or “closed” (bound to NAD^+^ and substrate) conformation. Upon substrate binding, an ∼18° rotation of the hinge brings together the cofactor- and substrate-binding domains, followed by a rotation of the dimers by ∼14°C (Yin et al., 2000; Yang et al., 2003). Once the reaction has occurred, the enzyme reverts to an open conformation and the product is released (Wang et al., 2005). These transitions from open-to-closed conformation would facilitate the steps of the enzymatic reaction as well as the diffusion of substrates (Wang et al., 2005).

### Enzymatic Activity Regulation

The enzymatic reaction for the reversible catalysis of SAH, first described in 1979 (Palmer and Abeles, 1979), was later supported by analysis of the mammalian structure of AHCY (Kusakabe et al., 2015). At the mechanistic level, AHCY mediates a nucleophilic enzymatic cascade enabled by redox steps (Walsh and Moore, 2019). The reaction is initiated (both *via* the forward breakup or reverse synthesis of SAH) by an oxidation of SAH or adenosine substrates by the enzyme-bound NAD^+^. The oxidated intermediate is then cleaved to release homocysteine (Hcy) or water (depending on the substrate) and then is subsequently reduced by NADH to form the final product (adenosine or SAH). In thermodynamic equilibrium, the reaction is largely favored toward the synthesis of SAH *in vitro*, but efficient removal of adenosine and homocysteine enables the net breakup of SAH *in vivo* (Palmer and Abeles, 1979; Kusakabe et al., 2015; Walsh and Moore, 2019).

Proteomic analysis has revealed that mammalian AHCYs are acetylated at lysines 401 and 408 within their *C*-terminal tail (Choudhary et al., 2009). *In vitro*, bi-acetylated-K401/408 human AHCY displays a threefold reduction of the catalytic constant and two-fold increase of SAH K*m* (Wang et al., 2014). Comparative analyses between unmodified and acetylated structures of AHCY indicate a local hydrogen-bound alteration in the vicinity of the modified lysine residues, indicating that slight structural changes in AHCY might have a significant functional impact on its catalytic activity. Two additional modifications, based on the conjugation with short fatty acid derivatives on lysine residues, have recently been reported: the 2-hydroxyisobutyrylation (hib) of lysine (K) 186, and the β-hydroxybutyrylation (bhb) of several lysines (20, 43, 188, 204, 389, and 405) (Huang et al., 2018; Koronowski et al., 2021). In particular, forced K-bhb inhibits AHCY activity in mouse embryonic fibroblast (MEFs) and mouse liver (Koronowski et al., 2021). Enzymatic assays from cells ectopically expressing mutants show that K188R, K389R, and K405R substitutions compromise AHCY activity (Koronowski et al., 2021). In addition to lysine modification, mouse AHCY is posttranslationally modified with an *O*-linked β-*N*-acetylglucosamine sugar (*O*-GlcNAcylation) at threonine 136 (Zhu et al., 2020). The oligomerization capacity of AHCY (and therefore, its enzymatic activity) is reduced by mutation of threonine 136 to alanine (T136A), as well as by pharmacological inhibition of glycosylation (Zhu et al., 2020). Importantly, mouse embryonic stem cells (mESCs) expressing AHCY-T136A mutant display a reduced proliferation and low alkaline phosphatase staining (Zhu et al., 2020), suggesting that AHCY glycosylation is important to balance self-renewal and pluripotency in mESCs. Thus, posttranslational modifications in AHCY can impact the structure of the enzyme, thereby modulating its enzymatic activity and its biological role. In line with this, mass spectrometry-based proteomic discovery has revealed an accumulation of other posttranslational modifications (i.e., phosphorylation, acetylation, and ubiquitylation) in the vicinity of the non-catalytic, movable hinge domain and the *C*-terminal domain (source www.Phosphosite.com) (Figure 1), pointing toward their potential roles as AHCY regulatory domains.

Several resolved AHCY structures contain monovalent cations, which would facilitate the catalytic activity. For instance, the resolved structure of the mouse AHCY has sodium cation allocated in the *C*-terminal hinge region (Kusakabe et al., 2015), contributing to the recognition of the substrate, similarly as the plant enzyme (Brzezinski et al., 2008). In addition, the AHCY from *Pseudomonas aeruginosa* binds potassium cations, and kinetic studies indicate that potassium stimulates AHCY enzymatic activity and ligand binding (Czyrko et al., 2018). Conversely, divalent cations, such as zinc and copper, have been shown to inhibit AHCY activity. Structural studies of AHCY from *P. aeruginosa* indicate that zinc ions are coordinated with AHCY homotetramers near the gate of the active sites, thus preventing its accessibility (Czyrko et al., 2018). Even though a copper ion is not required for its protein stabilization or catalytic activity, AHCY is a strong copper binder both *in vitro* and *in vivo*, with a K*d* of about 1 × 10^–12^ for free-copper, and K*d* of about 1 × 10^–17^ in the presence of EDTA (Bethin et al., 1995; Li et al., 2004). *In vitro*, copper is a non-competitive inhibitor for the substrates, facilitating the dissociation of NAD^+^ in a concentration-dependent manner (Li et al., 2007). Although Cu-AHCY structures have not been resolved, computational predictions as well as the mechanism of inhibition suggest that Cu^2+^ binds the central core of the AHCY tetramer, preventing subunit interaction and/or decreasing NAD^+^ affinity (Li et al., 2007). Both AHCY’s capacity to bind copper, which is comparable with albumin (Masuoka et al., 1993), and its high abundance in the liver, where it comprises 0.5% of total hepatic cytosolic protein (Bethin et al., 1995), indicate that AHCY could contribute significantly to the hepatic copper metabolism and copper-associated human syndromes, such as Wilson disease. All these data suggest that cation-AHCY interaction may have significant relevance and that the biological and pathological cross-talk between AHCY and cations needs further exploration.

*In vitro* binding experiments have shown that bovine AHCY endows two adenosine binding sites, and their usage depends on the enzyme-bound NAD^+^/NADH ratio (Kloor et al., 2003). With a low affinity, adenosine binds the AHCY-NAD^+^ at the catalytic domain while, with high affinity, adenosine binds the enzymatically inactive AHCY-NADH at the cofactor domain (Kloor et al., 2003). Despite the importance of NAD^+^ as a cofactor, whether an intracellular fluctuation in NAD^+^/NADH concentrations influences AHCY activity or its adenosine binding *in vivo* remains unknown.

## Biological and Functional Roles of AHCY

### AHCY Function in Embryonic Development

Considering its strong conservation during evolution and its ability to affect the methylation potential in cells, the functionality of AHCY has been explored in multiple organisms.

The *Arabidopsis thaliana* genome harbors two AHCY paralogs (*AtSAHH1* and *AtSAHH2*) with non-redundant functions. While AtSAHH2 seems functionally dispensable in plants (Rocha et al., 2005), a loss-of-function mutation in AtSAHH1 is embryonic lethal and impairs the DNA methylation-dependent gene silencing (Furner et al., 1998). Moreover, genetic and pharmacological reduction of AtSAHH1 activity results in developmental abnormalities (i.e., slow growth and reduced fertility) (Furner et al., 1998; Rocha et al., 2005; Wu et al., 2009). At the molecular level, AtSAHH1 mutant plants display a general DNA hypomethylation and alterations in the expression of key developmental genes, a defect attributed to direct inhibition of plant DNA methyltransferases or histone methyltransferases required to sustain genome methylation (Rocha et al., 2005; Mull et al., 2006; Jordan et al., 2007; Ouyang et al., 2012). Similarly, tobacco plants with reduced expression or pharmacological blockade of AHCY show defects in growth and flowering, reduced DNA methylation on repetitive elements, and ectopic expression of genes critical for flower development in different organs (Tanaka et al., 1997; Fulnecek et al., 2011). Finally, the tomato genome contains three related genes encoding for highly conserved SAHH that display functional redundancy, in contrast to those in *A. thaliana* (Li et al., 2015). Simultaneous silencing of all three genes inhibits vegetative growth (Li et al., 2015). Overall, these studies indicate the essentiality of AHCY for proper plant development and growth.

The mouse genome harbors the *Ahcy* gene in chromosome 2, and at least five intron-less genomic sequences similar to the complementary DNA (cDNA) in chromosomes X, 1, and 16 (from 86 to 99.7% identity). These latter likely correspond to pseudogenes (based on the alignment of mouse *Ahcy* cDNA-CCDS16942.1, using BLAT; Kent, 2002), and at least one (in chr16) encodes for a potentially complete wild-type form of AHCY protein. Phenotypic analysis of a large knockout mouse line collection indicates that homozygous deletion of *Ahcy* is embryonic lethal before E9.5 (Dickinson et al., 2016), and chromosomal microdeletions (of about 100 kb) encompassing the *Ahcy* gene in mice cause recessive lethality at the blastula stage (Miller et al., 1994). Several pieces of evidence point to the essential role of *Ahcy* during early mouse embryo development at the pre-implantation stage. First, during the first 4.5 days post-coitum, *Ahcy* expression increases gradually from the zygote to blastula stages, following the acquisition of the pluripotency (Aranda et al., 2019). Second, the pharmacological treatment of mouse embryos with AHCY inhibitors severely compromises the blastula progression (Miller et al., 1994; Aranda et al., 2019). Third, specific depletion of *Ahcy* drastically reduces the proliferation of mESCs derived from blastocyst (Aranda et al., 2019), and a prolonged period of *Ahcy* depletion promotes spontaneous mESC differentiation (Zhu et al., 2020). Further generation of inducible or tissue-specific Ahcy knockout mice models would be of great interest to dissect its role during development or adult tissue homeostasis.

### AHCY Function in Cellular Stress

Metabolomics analysis of young and old *Drosophila melanogaster* indicates that aging in flies correlates with a remarkable accumulation of SAH. Conversely, fly strains naturally selected for their longevity display reduced levels of SAH (Parkhitko et al., 2016). Reduction of *dAhcy* by RNA interference in the whole body or in a neural-specific manner drastically increases SAH levels and reduces lifespan. Conversely, the suppression of two enzymatically inactive paralogs of *dAhcy* (*dAhcyL1* and *dAhcyL2*) increase lifespan, presumably because they act as dominant-negative *dAhcy* interactors (Parkhitko et al., 2016). In zebrafish, loss-of-function mutations in *Ahcy* are lethal, causing defects in exocrine pancreas development (Yee et al., 2005). In addition, mutants display hepatic steatosis (accumulation of lipids in hepatocytes) and liver degeneration early at larvae stage (Yee et al., 2005; Matthews et al., 2009), two common features observed in human patients with AHCY deficiency (see below). These studies suggest that AHCY plays a major role in controlling cellular homeostasis and tissue damage in animals. Although the mechanisms underlying increased aging and tissue degeneration in AHCY-deficient animals have not been experimentally determined, they are likely due to: (*i*) a reduced potential of the transmethylation reaction for specific substrates; (*ii*) increased oxidative stress, as a consequence of a reduction in the flux homocysteine into the transsulfuration pathway for glutathione production; and/or (*iii*) replication stress, caused by decreased availability of adenosine for nucleotide production.

In agreement with its potential role in cellular toxicity, a knockdown of AHCY in the hepatocellular carcinoma cell line HepG2 results in attenuation of cell cycle progression and increased DNA damage (Beluzic et al., 2018). The cytotoxic effects can be reversed by adenosine supplementation, thus suggesting that mild inactivation of AHCY may cause cellular stress in liver cells, due to adenosine depletion (Beluzic et al., 2018).

### Molecular Effects on DNA, RNA, and Histone Methylation

**FIGURE 2:**
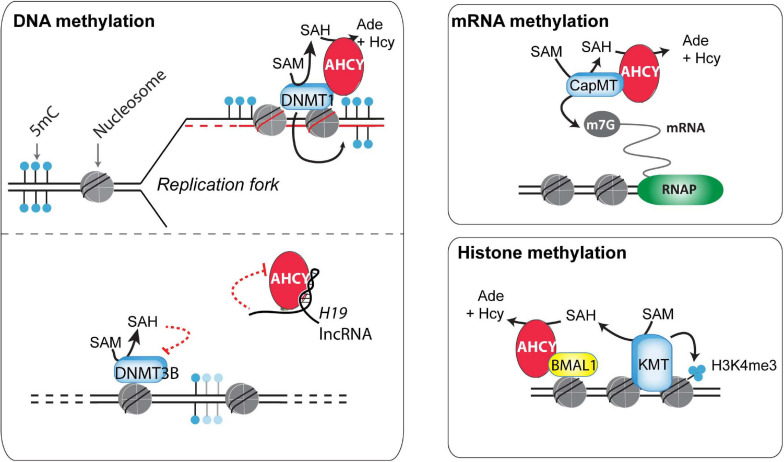
Potential mechanisms of action of AHCY in mammals. Different mechanisms of action have been proposed for the control of DNA, RNA, and histone methylation (discussed in the main text).

DNA methylation is a well-studied and paradigmatic epigenetic mark (Bonasio et al., 2010). Once deposited, the 5-methylcytosine mark can self-perpetuate by a well-known molecular mechanism, influencing genome functionality. DNA methyltransferase 1 (DNMT1), the enzyme responsible for the maintenance of DNA methylation, recognizes hemimethylated DNA during replication and establishes the methyl group in the newly synthesized DNA (Bonasio et al., 2010). This process is assisted by several accessory proteins, including the multidomain protein UHRF1 (Blanchart et al., 2018). In a proteomic survey during the cell cycle of HeLa cells, AHCY was found as a partner of DNMT1 (Ponnaluri et al., 2018) (Figure 2). *In vitro*, methyltransferase assays indicated that AHCY enhances the DNMT1 activity, and *in vivo*, AHCY overexpression induces a pervasive increase in DNA methylation in HEK293 cells (Ponnaluri et al., 2018). As this suggests that AHCY is a rate-limiting factor for DNA methylation maintenance, its role during epigenomic reprogramming throughout embryo development and/or in disease progression, such as cancer, must now be investigated.

In addition to maintaining DNA methylation, AHCY also exerts a role in regulating *de novo* DNA methylation (Zhou et al., 2015). This role is proposed to be mediated throughout the AHCY inhibition by the developmentally regulated long non-coding RNA *H19*. *In vitro*, U-rich nucleotide elements present in *H19* reduce the ability of recombinant AHCY to breakdown SAH *in vitro* (Zhou et al., 2015). *In vivo*, knockdown of *H19* increases the cellular activity of AHCY in myotubes differentiated from mouse myoblasts. As a consequence, *H19* knockdown induces extensive changes in the pattern of genome methylation in myotubes, with specific DNMT3B-target genes gaining DNA methylation in an AHCY-dependent manner. Overall, this indicates that, consistent with its function, AHCY deficiency impacts both in DNA methylation maintenance as well as in *de novo* methylation, thus profoundly influencing the epigenome of cells (Figure 2).

RNA methyltransferases are highly sensitive to the reduction of the SAM:SAH ratio (Clarke, 2001). Indeed, pharmacological treatment with 3-deazaadenosine, a specific AHCY inhibitor, induces reduction of methyl RNA levels in mouse macrophage and osteosarcoma cells, including loss of the N6-methyladenosine (m6A) (Backlund et al., 1986). In mouse embryonic fibroblasts, this m6A reduction upon AHCY inhibition is functionally linked to a delay in processing RNAs from circadian rhythm genes, thus impacting their oscillatory behavior response (Fustin et al., 2013). Extending this observation, Fustin et al. (2020) have recently found that targeting AHCY activity with 3-deazaneplanocin A (DZnep) produces a loss in the oscillatory transcriptional response of circadian-associated genes in different organisms (from unicellular algae to humans). The loss of the oscillatory transcriptional activation of the *Hes7* gene during somitogenesis on DZnep-treated mouse embryos suggests that methylation flux, directed by AHCY, is a universal regulator of biological circadian rhythms in metazoans (Fustin et al., 2020).

Global steady-state levels of cap methylation on mRNA (7-methylguanosine, m7G) are seemingly unaffected upon AHCY inhibition in mouse embryonic fibroblasts (measured as the ratio m7G methylated/unmethylated mRNA) (Fustin et al., 2013). However, considering the tight association of m7G with mRNA stability, the uncapping of pre-mRNA could be linked with an increase in pre-mRNA degradation, and, therefore, the impact of AHCY inhibition in m7G regulation might be overlooked. Indeed, several experimental evidences show that increased m7G demands are dependent on AHCY functionality in some instances. In developing Xenopus, xAHCY, which remains nearly cytosolic until the blastula stage, starts to accumulate in the nucleus of differentiating cells, which experience a boost in transcriptional activity during gastrulation (Radomski et al., 1999). In Xenopus oocytes, xAHCY displays a nuclear accumulation on active transcribing loops on lampbrush chromosomes (Radomski et al., 1999) and interacts with mRNA (guanine-7-) methyltransferase through its proximal *N*-terminal region (Radomski et al., 2002). Furthermore, its pharmacological inhibition compromises both methylation and synthesis of nuclear RNA (Radomski et al., 1999, 2002). Similarly, in human and murine cells, an increased demand for m7G induced upon MYC activation depends on AHCY activity (Fernandez-Sanchez et al., 2009). Upon AHCY knockdown, the MYC-induced m7G is compromised and correlates with reduction in Myc-induced core effects in protein synthesis, cell proliferation, and transformation. Interestingly, *AHCY* is a direct MYC target and it is transcriptionally upregulated in an MYC-dependent manner in numerous cell types (Fernandez-Sanchez et al., 2009). Thus, AHCY appears to be a key response molecule in controlling the molecular and functional effects of Myc.

Based on its role on controlling RNA methylation, AHCY has been long considered a target for antiviral strategies (Bader et al., 1978; De Fazio et al., 1990; Snoeck et al., 1993; Masuta et al., 1995; Daelemans et al., 1997; Chiang, 1998; Daelemans et al., 1998; Huggins et al., 1999; Bray et al., 2000; De Clercq, 2005, 2015; Yoon et al., 2019; Shin et al., 2020). The antiviral properties of AHCY inhibition are based on the metabolic accumulation of SAH, which directly inhibits viral or host RNA MTases required for successful viral spreading (Daelemans et al., 1998; De Clercq, 2005; Liu and Kiledjian, 2006; Leyssen et al., 2008). AHCY inhibitory compounds show a broad-spectrum antiviral activity against RNA viruses, including rhabdo-, filo-, arena-, paramyxo-, reo-, and retroviruses. *In vivo* efficacy of AHCY inhibitors is remarkable, as a single dose of either of the three distinct AHCY inhibitors 3-deazaneplanocin A, 3-deazaaristeromycin, or 3-deazaadenosine protects mice against a lethal infection of the Ebola virus (subtype Zaire) (Huggins et al., 1999; Bray et al., 2000, 2002). Recently, the list of AHCY-sensitive viruses has increased, as the novel potent AHCY inhibitors of 6′-fluorinatedaristeromycin analogs have notable activity against emerging positive-stranded RNA (+RNA) viruses, such as the severe acute respiratory syndrome coronavirus (SARS-CoV1), Middle East respiratory syndrome coronavirus (MERS-CoV), chikungunya virus (CHIKV), and Zika virus (ZIKV) (Yoon et al., 2019; Shin et al., 2020). These results have placed 6′-fluorinatedaristeromycin as a drug candidate against COVID-19.

The methylation of core nucleosome histones, which are the most abundant eukaryotic nuclear proteins, influences protein–protein interactions, chromatin structure, and gene expression (Espejo et al., 2020). The potential role of the nucleosome as a SAM consumer is high, as methylation of ∼0.1% of all histone residues can consume the complete cellular SAM pool in mammalian cells (Ye and Tu, 2018). Arginines and lysines can be extensively methylated on histone proteins, with the histone 3 lysines 4, 9, 27, 36, and 79 (H3K4, H3K9, H3K27, H3K36, and H3K79, respectively) being the most well-characterized (Zhao and Garcia, 2015). Among the different potential effects of AHCY blockade over histone methylation, the global and local di- and tri-methylation of lysine 4 on histone H3 (H3K4me2/3) appears the most sensitive (Fustin et al., 2013; Greco et al., 2020; Zhu et al., 2020). Recently, Greco et al. (2020) found that the oscillatory increase of H3K4me3 in circadian rhythm-related genes is compromised after pharmacological inhibition of AHCY in mouse embryonic fibroblasts. At the molecular level, AHCY interacts with BMAL1, a key circadian transcription factor, and regulates the oscillatory recruitment of BMAL1 to chromatin, thus impacting the amplitude of the transcriptional oscillations of circadian rhythm-related genes (Greco et al., 2020) (Figure 2). The authors suggest that the dimer AHCY-BMAL1 cooperates with histone methyltransferases for gene activation and that AHCY removes excess of SAH to facilitate local histone methylation demands during the transcription peaks.

## Impact of AHCY in Human Diseases

### AHCY Involvement on Rare Metabolic Disorders

Wilson disease (WD) is a rare, inborn disorder with severe hepatic and neurological dysfunctions due to the pathological accumulation of copper (Czlonkowska et al., 2018). The clinical symptomatology in WD is highly variable for age of onset (ranging from 5 to 35 years) and severity, varying from nearly asymptomatic with mild changes in personality to acute liver failure or parkinsonian-like effects. Although over 300 disease-causing mutations have been reported in the copper transporter *ATP7B*, the genotype–phenotype correlation in WD is very poor (Czlonkowska et al., 2018). This suggests the existence of other genetic risk factors influencing WD. As AHCY is a strong copper binder, and copper inhibits AHCY activity in a dose-dependent and non-competitive manner (see above), a pathological link between copper accumulation and the inhibition of AHCY has been proposed. Indeed, the pathological accumulation of copper in the Jackson toxic milk mutant mice (tx-J), which carry a missense mutation in the *Atp7b* gene, reduces AHCY expression (Bethin et al., 1995; Li et al., 2004, 2007). In this WD murine model, deficit in AHCY associates with an increase of SAH levels, reduction in the SAM:SAH ratio, and DNA hypomethylation (Medici et al., 2013, 2014, 2016). Interestingly, feeding tx-J mice with diets supplemented with betaine or choline normalizes the SAM:SAH ratio, global DNA hypomethylation, and the expression of hepatic genes related to methionine homeostasis (Medici et al., 2013, 2014, 2016). These data provide a link between hepatic copper accumulation and the function on AHCY in the liver.

In humans, at least 204 missense *AHCY* variants are annotated in public databases^1^ or reported in the literature. Twelve of these variants are linked with a rare autosomal recessive disorder in the methionine metabolism of hypermethioninemia (R49C, R49H, A50T, T57I, G71S, D86G, A89V, E108K, T112stop, Y143C, V217M, and Y328D), characterized by a deficiency of AHCY (OMIM: 180960) (Baric et al., 2004, 2005; Buist et al., 2006; Vugrek et al., 2009; Honzik et al., 2012; Stender et al., 2015; Strauss et al., 2015; Vivante et al., 2017; Bas et al., 2020; Grudzinska Pechhacker et al., 2020). Patients typically display severe hepatic, muscle, and cognitive dysfunction, including multiorgan failure followed by death soon after birth (Tehlivets et al., 2013; Stender et al., 2015; Judkins et al., 2018). In addition to this most frequent and severe version, a mild version of the disease has been reported in one family, who remain nearly asymptomatic during childhood but presented severe hepatic disorders during adulthood (Stender et al., 2015). Dietary interventions that reduce methionine uptake have shown variable benefits for patients, questioning its therapeutic advantage in AHCY deficiency syndrome. For one 40-month-old child for whom dietary therapy was ineffective, liver transplantation restored metabolic parameters and reversed psychomotor and cognitive deficits after 6 months (Strauss et al., 2015). However, the lack of further assessment on the course of the disease, and the difficulties of finding a rapidly compatible donor soon after diagnosis, would argue against liver transplantation as a general indication for AHCY deficiency. Thus, finding an effective therapy is urgently needed for treating AHCY deficiency.

### Role of AHCY in Cancer

Adenosylhomocysteinase was initially defined as a tumor suppressor (Leal et al., 2008). In agreement, AHCY-deficient mouse embryonic fibroblasts have a higher proliferative capacity and the ability to escape from replicative senescence (Leal et al., 2008). At a mechanistic level, an AHCY knockdown overcomes the p53-induced cell cycle arrest and abrogates p53-dependent transcriptional activity (Leal et al., 2008). In agreement with a tumor suppressor role, *AHCY* mRNA expression was found to be reduced in a large number of different solid tumor samples as compared with non-tumor paired tissue (Leal et al., 2008).

However, AHCY’s function as a tumor suppressor seems to be cell type specific, as AHCY inhibition is also linked to anti-migratory and anti-invasive activity in breast cancer cells (Kim et al., 2013; Park et al., 2015) and to enhanced apoptosis in high aggressive neuroblastoma (Westermann et al., 2008; Chayka et al., 2015). In neuroblastoma, AHCY expression is elevated in MYCN-amplified tumor samples and neuroblastoma cell lines (Westermann et al., 2008; Chayka et al., 2015). Interestingly, AHCY knockdown or drug-mediated inhibition induces an increase in apoptosis specifically on MYCN-amplified neuroblast cells (Chayka et al., 2015), thus showing a specific synthetic lethality and making AHCY inhibition a promising therapeutic possibility for personalized treatment of high-risk neuroblastoma.

## Conclusion

Methylation is one of the most ubiquitous chemical reactions in living organisms (Clarke, 2001). While some methyltransferases retain most of their activity at different SAM:SAH ratios, such as the glycine *N*-methyltransferase, others are more susceptible to SAH elevation, including tRNA or arginine methyltransferases (Clarke, 2001; Richon et al., 2011; Zhang and Zheng, 2016). Thus, considering the large number of SAM-dependent MTases, which account for nearly 1% of the human protein-coding genes (∼200), their different functionalities, and their distinct sensitivity to SAH accumulation (Clarke, 2001; Petrossian and Clarke, 2011; Richon et al., 2011; Zhang and Zheng, 2016), it is difficult to foresee which is the most relevant methyltransferase affected upon AHCY inhibition. Thus, after AHCY blockade, the influence of one molecular outcome (e.g., loss of DNA methylation, RNA methylation, or protein methylation) in a particular molecular process will depend very much on the following: (*i*) the biological context (e.g., cell type, stimulus); (*ii*) the molecular event involved in this biological process (e.g., transcription); (*iii*) the most relevant methyltransferase involved; and (*iv*) their relative sensitivity to SAH accumulation.

Also, as the result of the AHCY activity, the methionine cycle can supply the production of adenosine for nucleotide biosynthesis and for homocysteine, the precursor for the biosynthesis of glutathione in higher eukaryotes (Ye and Tu, 2018). Therefore, in addition to increasing the methylation potential, SAH turnover could be essential to cope with replicative-associated oxidative stress, thus expanding the repertoire of the biological process where AHCY plays a role.

Methylations occur in all subcellular compartments. The intracellular concentrations of SAM (∼10 μM) is at the range of the K*m* of the SAM-dependent MTases, but it can fluctuate 10–100-fold in normal physiological conditions (Clarke, 2001; Ye and Tu, 2018). The nuclear heterogeneity at the transcriptional level and during replication, as well as the existence of phase-separated membrane-less subnuclear compartments (Erdel and Rippe, 2018), likely produce large differences in local fluxes of SAH due to localized transmethylation reactions, such as DNA methylation maintenance at replication sites, or mRNA cap methylation at transcriptionally active chromatin regions (Espejo et al., 2020). Hence, the local recruitment of AHCY seems required to sustain effective transmethylation reactions in time and space. We and others have found that AHCY is recruited to chromatin at specific sites (Aranda et al., 2019; Greco et al., 2020). Our recent discoveries unveil a novel chromatin function of AHCY in controlling gene expression, which is in line with recent data identifying the association of other metabolic enzymes with chromatin (Li et al., 2018). However, our discoveries also present new questions at the mechanistic level: How is AHCY recruited to specific chromatin regions in mammals? How does AHCY control local gene expression? Further studies on protein–protein interactions within chromatin will clarify the molecular mechanisms underlying AHCY’s role in gene regulation. This research direction might also have an impact in AHCY-related pathologies as changes in the nucleocytoplasmic distribution have been reported in several pathological AHCY variants (Grbesa et al., 2017). Therefore, the potential pathogenicity caused by mutants that cause aberrant nuclear localization of AHCY might be linked with its chromatin-related functions.

Hundreds of missense polymorphisms are present in the human *AHCY* gene across the population (see text footnote 1), some of which have a similar allelic frequency as those identified as pathological variants. This, together with the asymptomatic evolution of some patients with defects in AHCY activity until fertile adulthood (Stender et al., 2015), suggest that AHCY deficiency could be underdiagnosed, and that the impact of AHCY in human pathologies could be underestimated in the general population.

In 2004, AHCY deficiency was first identified in a 5-month-old Croatian infant with delayed psychomotor development, hypotonia, attention defects, and poor head control since birth, who also presented with elevated levels of aminotransferase, methionine, SAH, and SAM in serum (Baric et al., 2004). Since then, additional patients harboring *AHCY* mutations have been described with similar clinical traits. However, only few associated mutations have been characterized at the molecular level. In addition to the pathological variants that strongly affect AHCY activity, non-pathologic polymorphic isoforms of AHCY found in different populations display reduced thermal stability of the protein and enzymatic activity (Fumic et al., 2007). More recently, genetic variations of *AHCY* have been linked to different prognosis of children with neuroblastoma (Novak et al., 2016), thus providing evidence of the potential use of AHCY variants as a molecular biomarker. Considering the pleiotropic effects of AHCY, timely identification of AHCY deficiency may allow for targeted intervention to hinder the progression of related diseases. A systematic analysis of all potentially deficient AHCY variants would provide molecular tools for genetic counseling, as well as for improving the prediction, diagnosis, and clinical outcome of AHCY deficiencies.

The aggressiveness of neuroblastoma cancer subtypes seems to rely on AHCY functionality (Westermann et al., 2008; Chayka et al., 2015). This exciting result places AHCY inhibitors as potential agents to target metabolic dependencies in some cancer types. Supporting this notion, tumor-initiating cells from non-small-cell lung cancer (NSCLC) adenocarcinoma display metabolic reprograming and rely on methionine to fuel their tumorigenicity (Wang et al., 2019). Interestingly, pharmacological alterations of the methionine cycle, by blocking AHCY with a specific inhibitor, reduce the colony- and tumor-forming abilities of tumor-initiating cells (Wang et al., 2019). Understanding the basis of AHCY-directed metabolic alterations is therefore essential to gain insights into the sensitivity of specific cancer types to therapeutic interventions based on AHCY inhibition and is a prerequisite for developing effective therapies.

## Author Contributions

SA wrote the manuscript and created the figures, together with PV and LD. All authors contributed to the article and approved the submitted version.

## Conflict of Interest

The authors declare that the research was conducted in the absence of any commercial or financial relationships that could be construed as a potential conflict of interest.

## Footnotes

1 https://gnomad.broadinstitute.org/
